# Eliminating mother-to-child transmission of hepatitis B virus: practice and progress in Baoan, a national pilot district of China

**DOI:** 10.1186/s12889-023-17500-y

**Published:** 2024-01-02

**Authors:** Ya-Li Luo, Shuang Gao, Quan-Fu Zhang, Xian Liu, Ding-Yan Lv, Jia-Hong Chen, Wei Wang

**Affiliations:** 1 Department of Health Care, Shenzhen Baoan Women’s and Children’s Hospital, Shenzhen, Guangdong China; 2 Department of Maternal Health Care, Guangdong Women and Children’s Hospital and Health Institue, Guangzhou, Guangdong China; 3 Office of Hospital Director, Shenzhen Baoan Women’s and Children’s Hospital, Shenzhen, Guangdong China; 4 Department of Anesthesiology and Surgery, Shenzhen Baoan Women’s and Children’s Hospital, Shenzhen, Guangdong China

**Keywords:** Elimination of mother-to-child transmission, Hepatitis B virus, Screening, Antiviral therapy, Post-vaccination serological testing

## Abstract

**Background:**

While mother-to-child transmission (MTCT) of hepatitis B virus (HBV) remains a significant challenge in China, research investigating the effectiveness of the September 2017 pilot program to eliminate MTCT of HIV, syphilis, and HBV is limited. Baoan district, which has a higher-than-average rate of hepatitis B infection among pregnant women and strong support from the government, was one of six national pilot districts selected for the program. Therefore, this study aims to assess the progress and implementation of the elimination of MTCT of HBV in Baoan district over a period of 5 years.

**Methods:**

Data was collected from the national information system for the prevention of MTCT, registration forms, and follow-up forms of pregnant women and their live births from 2018 to 2022. Joinpoint models were used to analyze changing trends over time, calculating annual percentage change (APC) and the corresponding 95% confidence interval (95%CI). Multivariate logistic regression models were used to analyze risk factors for HBV MTCT.

**Results:**

From 2018 to 2022, the coverage of HBV screening during pregnancy increased from 98.29 to 99.55% (APC = 0.30, *P* = 0.012). The coverage of HBV early screening within 13 gestational weeks increased from 40.76 to 86.42% (APC = 18.88, *P* = 0.033). The prevalence of maternal HBV infection declined by an APC of − 3.50 (95% CI -6.28 ~ − 0.63). The coverage of antiviral therapy among high-risk pregnant women increased from 63.59 to 90.04% (APC = 11.90, *P* = 0.031). Coverage for timely administration of hepatitis B immunoglobulin, hepatitis B birth dose vaccine, and three-dose hepatitis B vaccination remained consistently above 97.50%. The coverage of post-vaccination serological testing (PVST) in high-risk infants was 56.15% (1352/2408), and the MTCT rate of HBV was 0.18%. Mothers with high-school education or below (OR = 3.76, 95% CI 1.04 ~ 13.60, *P* = 0.04) and hepatitis B e antigen (HBeAg) positivity (OR = 18.89, 95% CI 1.98 ~ 18.50, *P* = 0.01) had increased MTCT risk.

**Conclusions:**

The implementation of comprehensive prevention strategies in Baoan district, including screening, treatment, and immunoprophylaxis, has proven effective in maintaining the MTCT of HBV at an extremely low level. However, it remains crucial to raise public awareness, specifically on the importance of improving the coverage of PVST for infants exposed to HBV.

**Supplementary Information:**

The online version contains supplementary material available at 10.1186/s12889-023-17500-y.

## Background

Hepatitis B virus (HBV) infection is a major global public health issue, affecting an estimated 257 million people worldwide [1]. The prevalence of HBV infection varies greatly among different regions and countries, with the highest rates found in East Asia and sub-Saharan Africa. In these regions, up to 10% of the general population may be infected with HBV, compared to less than 2% in North America and Europe [2].

HBV infection is known to cause liver cirrhosis, liver cancer, and liver failure, resulting in approximately 780,000 global deaths annually [1]. Additionally, those infected with HBV have an increased risk of developing chronic infection, leading to long-term complications and further transmission. In response, the World Health Organization (WHO) has set a global target of reducing the incidence of HBV in children to less than 0.1% by 2030. The successful achievement of this target will require a combination of strategies, such as the implementation of safe and effective vaccines, improved screening and treatment access, and enhanced prevention tactics [3, 4].

Approximately 93 million Chinese are estimated to be infected with HBV, which is about a third of the global HBV burden [1]. The prevalence of HBV infection in the Chinese general population is approximately 7% [5], while chronic HBV carriers account for roughly 6.17% of pregnant women [6]. Mother-to-child transmission (MTCT) is the predominant route of HBV transmission in endemic areas, accounting for about 40–50% of all new HBV infections in China [7].

China’s efforts to combat HBV infection began in 1992 when the hepatitis B vaccine was included into the Expanded Program on Immunization (EPI). The integration of free hepatitis B immunization in 2002 further increased vaccination coverage [8, 9]. These programs have led to a significant reductions in the prevalence of HBV infection among the general population (from 9.2 to 7.2% from 1992 to 2006) and in children under 5 years old (from 0.96 to 0.32% from 2006 to 2014) [9].

In 2010, prevention of MTCT (PMTCT) work was included in the national major public health service project, expanding from solely preventing HIV to integrating strategies to prevent the transmission of HIV, syphilis, and HBV, pioneering a new global effort in PMTCT. In 2015, this important initiative was extended nationwide, ensuring that pregnant women are screened for HBV through hepatitis B surface antigen (HBsAg) and that all HBV-exposed newborns receive free administration of hepatitis B immunoglobulin (HBIG) and hepatitis B birth dose vaccine (HepB-BD) within 24 hours of birth [10, 11]. In addition, the Chinese government endorsed the 2018–2030 Asia Pacific Regional Framework for triple elimination of MTCT (EMTCT) of HIV, syphilis, and HBV in 2017, to further improve the public health system’s efforts to tackle HBV infection in China and the Asia-Pacific region [3].

Although China has made significant progress in the PMTCT of HBV in recent years, numerous challenges persist. For instance, HBV DNA testing and antiviral therapy (ART) services for HBsAg positive pregnant women need to be enhanced, and the coverage of post-vaccination serological testing (PVST) for HBV-exposed children must be increased [12]. To effectively address these challenges, extensive measures need to be implemented and carefully evaluated, particularly in high-burden areas. In September 2017, the National Health and Family Planning Commission of China launched a pilot project in Zhejiang, Guangdong, and Yunnan provinces [13], with the aim of identifying effective models for the EMTCT of HIV, syphilis and HBV in China. Baoan district, located in Shenzhen, Guangdong province, was selected as one of six pilot counties/districts [14], and was subsequently chosen for comprehensive intervention with the goal of achieving “Zero” transmission of HBV from mother-to-child in Guangdong province [15]. Baoan was appointed as a pilot area primarily because of its high prevalence of HBsAg positive among pregnant women (9.06% during 2015–2019) [16]. Additionally, Baoan has exhibited strong policy support for the implementation of interventions for all pregnant women with HBV infection.

This paper aims to evaluate the effectiveness of interventions implemented in Baoan and assess their reference for HBV prevention and control in China. Through an analysis of outcomes from the pilot initiative, the paper seeks to identify strategies that could be adopted to further improve the EMTCT of HBV in other high-burden regions of the country.

## Methods

### Study design and setting

This cross-sectional retrospective study was conducted in Baoan district, China, including all 18 midwifery hospitals and over 140 community health service centers. The study used a hospital-based longitudinal analysis approach, recruiting pregnant women who gave live births from January 1, 2018 to December 31, 2022. In Baoan, only hospitals with midwifery qualifications provide delivery services. While most women deliver in these hospitals, there are some cases of out-of-hospital deliveries at home or during transportation. In such cases, the postpartum women are taken to midwifery hospitals for delivery registration. After discharge, newborns are managed by community health service centers for vaccination.

Baoan is the largest administrative districts (387.96 km^2^) and most populous (5.37 million at the end of 2021) among the 10 districts of Shenzhen, the first Special Economic Zone established under China’s reform and opening-up policy. Rapid urbanization in recent decades has propelled Shenzhen’s development, making it one of the most developed cities in China. Millions of migrants of reproductive age have been attracted to the city since the 1980s [17]. As a result, Baoan has experienced a significant increase in live births since 2000, rising from approximately 10,000 to almost 40,000 in 2022, largely due to the influx of migrant childbearing women. Consequently, Baoan presents an ideal setting to evaluate the implementation and effectiveness of intervention measures in a high-burden area with a large migrant population.

To ensure methodological transparency and reproducibility, this study adhered to the STROBE guidelines for reporting observational studies [18]. The sample size calculation was determined using the following formula:

$$n=\frac{Z_{\alpha /2}^2\times \left(1-p\right)\times p}{{\left(\delta p\right)}^2}$$

*p* was set at 1.2% based on the reported MTCT rate among HBV-exposed infants following immunization intervention in recent research conducted in China [19], with an allowable deviation of *δ* =0.15 and a significance level of *α* = 0.05. Using these parameters, a minimum study sample size of 14,057 mother-child pairs was calculated.

### Intervention package

In Baoan district, the intervention package is implemented sequentially, starting from the initial screening of pregnant women who tested positive for HBsAg, to the follow-up of the HBV-exposed children until they reached 12 months of age. The processes are as follows:

#### Screening

**Fig. 1:**
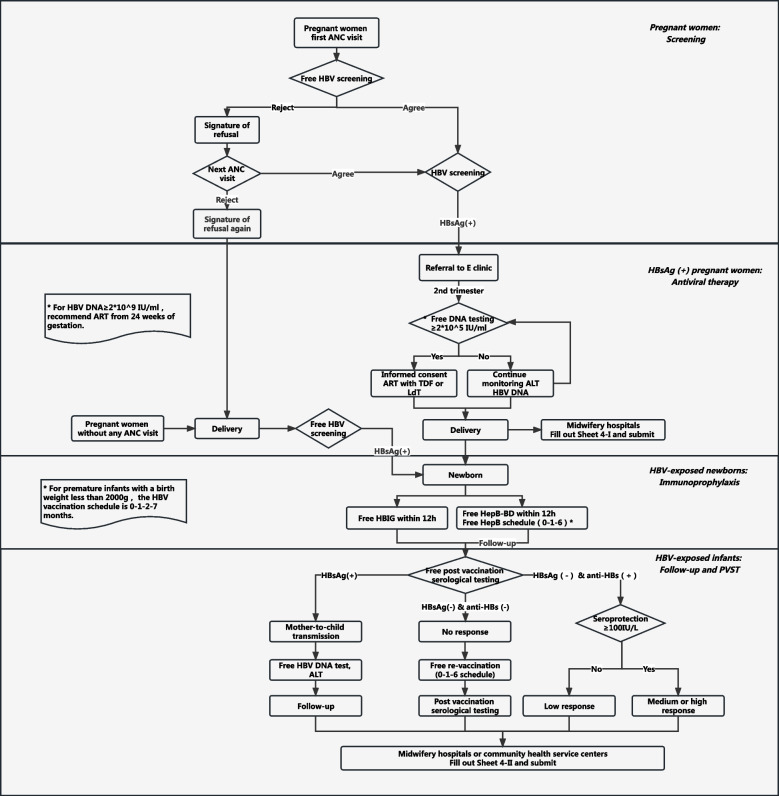
The latest intervention package (2021 edition) of HBV EMTCT in Baoan district

As illustrated in Fig. 1, all 18 midwifery hospitals were instructed to provide integrated counseling and free screening for HIV, syphilis, and HBV to pregnant women, either during their first ANC visit or at the time of delivery. Since May 2018, screening has been conducted prior to ANC registration to facilitate early identification of infection status (Supplementary Fig. S1). The HBV serological markers tested, which included HBsAg, antibody against HBsAg (anti-HBs), hepatitis B e antigen (HBeAg), antibody against HBeAg (anti-HBe), and antibody against hepatitis B core antigen (anti-HBc). were analyzed via Enzyme-linked immunosorbent assays (ELISA) in accordance with standard protocol.

#### Antiviral therapy

In November 2018, the EMTCT clinic (E clinic) was established in the obstetrics department of all midwifery hospitals in Baoan district. Pregnant women who tested positive for HBsAg were promptly referred to the E clinic for liver function and free HBV DNA testing. Following informed consent, high-risk women were required to undergo medication treatment at either 24 or 28 weeks of pregnancy. For most pregnant women, tenofovir disoproxil fumarate (TDF) was the preferred option. However, for those with kidney disease or severe osteoporosis, telbivudine (LdT) was recommended as an alternative treatment. Each midwifery hospital provided follow-up care throughout the pregnancy and submitted *Sheet 4-I Registration Form for pregnant women with HBV infection and their newborns* (Supplementary Table S1) to the Baoan Women’s and Children’s Hospital within 5 days of delivery.

#### Immunoprophylaxis

In keeping with the “the sooner, the better” immunoprophylaxis principle, Baoan district began implementing the” immediate immune next to bedside” strategy in January 2018. Delivery hospitals provided free HepB-BD and HBIG to newborns who had been exposed to HBV in the delivery room or operating room. Community health service centers provided the second and third vaccine doses, free of charge, at 1 month and 6 months after birth, respectively.

#### Follow-up and PVST

In December 2018, an Immunological Monitoring clinic (IM clinic) was set up in all midwifery hospitals in Baoan district to offer follow-up services for HBV-exposed infants who had completed the three-dose HepB vaccine series. These services included free HBsAg and anti-HBs testing and were provided until the infants turned 12 months of age. The respective hospitals completed and submitted *Sheet 4-II Follow-up Form for infants born to pregnant women with HBV infection* (Supplementary Table S2) to the Baoan Women’s and Children’s Hospital.

### Indicator evaluation

We adopted the indicators (Supplementary Table S3) outlined in the “*Guidance Manual for the Validation of Eliminating Mother-to-Child Transmission of HIV, syphilis, and hepatitis B (2022 edition)” d*eveloped by the National Center for Women and Children’s Health, China CDC) [20]. This manual is designed in line with the WHO’s “*Global guidance on criteria and processes for validation: elimination of mother-to-child transmission of HIV, syphilis and hepatitis B virus (2021)”* [21] and is intended for national validation. With these indicators serving as our benchmark, we assessed the availability of data and progress against the established indicators to facilitate the elimination validation process in Baoan.

It is worth noting that the management of pregnant women with HBV infection in Baoan district between 2018 and 2020 followed the *Management Algorithm for Interrupting Mother-to-Child Transmission of Hepatitis B Virus (2018 edition)* [22]. This algorithm defined high-risk women as those with a viral load equal to or greater than 2*10^6^ IU/ml, and recommended antiviral therapy during gestational weeks 24 to 28.

### Data collections

Data of ANC service, HBV screening and prevalence of maternal HBV infection were collected through the Information System of the National Integrated Prevention of Mother-to-Child Transmission of HIV, syphilis and hepatitis B (iPMTCT) Programme. This system reports data on women with the aforementioned infections who access ANC services from health care institutions and delivery hospitals on a monthly basis (Supplementary Table S4). Additionally, we extracted data for all live births during the same period (2018–2022) from the Annual report on Maternal Health of Baoan district to calculate the coverage of ANC. Quality control was performed by experts at the county and higher administrative levels, following the national protocol and guidelines [23].

For every case, we retrospectively reviewed and extracted data on maternal socio-demographic characteristics, HBV related screening and antiviral therapy, pregnancy outcomes, birth information, and follow-up information independently from *Sheet 4-I* and *Sheet 4-II* by two authors (Lv and Chen). We ensured that the data collected was accurate by rechecking for outliers and missing values, and supplementing them by reviewing the original medical records.

### Statistical analysis

*Pearson χ* ^2^ test or *Fisher’s* exact test was utilized to analyze variable distributions. Joinpoint Regression models were applied to determine changing trends of the study indicators over the years, calculating annual percentage change and 95% confidence intervals (APC, 95%CI). The risk of HBV MTCT was assessed using multivariate logistic regression models, with adjusted odds ratios presented alongside 95% confidence intervals (OR and 95%CI).

We established a database using EpiData 3.0 and conducted statistical analysis using Joinpoint Regression Program (Version 5.0.1 - April 2023, Statistical Methodology and Applications Branch, Surveillance Research Program, National Cancer Institute) as well as SPSS Version 21.0 (Chicago, IL, USA). We set statistical significance at a two-tailed *P*-value of < 0.05, in accordance with generally accepted research standards.

## Results

### Trends in HBV screening and prevalence among pregnant women

The ANC coverage in Baoan district maintained stable at over 98% from 2018 to 2022 (*P* = 0.290). A total of 218,707 pregnant women were recruited, of whom 216,018 (98.77%) received HBV screening during pregnancy, while the remaining individuals were screened at the time of delivery. The coverage of HBV early screening (within the first trimester) in pregnant women increased from 40.76% in 2018 to 86.42% in 2022, with an APC of 18.88 (95%CI 2.64 ~ 37.68, *P* = 0.033).

In total, 18,194 pregnant women were diagnosed with HBV infection, resulting in an overall prevalence of 8.34% (95%CI 8.22 ~ 8.45). The prevalence of maternal HBV infection showed a significant downward trend over the study period, decreasing from 8.76% (95%CI 8.51 ~ 9.01) in 2018 to 7.61% (95%CI 7.35 ~ 7.88) in 2022 (APC = -3.50, 95%CI -6.28 ~ − 0.63, *P* = 0.031) (Table 1).

**Table 1:** Trends in HBV screening and prevalence of maternal HBV infection in Baoan district

Year	Number live births (a)	Number of pregnant women received ≥ one ANC (b)	Coverage of ANC (b/a, %)	Number of pregnant women (c)	Number of HBV screening during pregnancy (d)	Coverage of HBV screening during pregnancy (d/c, %)	Number of HBV early screening in pregnant women (e)	Coverage of HBV early screening in pregnant women (e/c, %)	Number of pregnant women with HBV infection (f)	Prevalence of maternal HBV infection [f/c,% (95%CI)]
2018	48,966	48,106	98.24	48,553	47,724	98.29	19,791	40.76	4252	8.76 (8.51–9.01)
2019	50,321	49,403	98.18	49,838	49,068	98.45	33,344	66.90	4238	8.50 (8.26–8.75)
2020	41,930	41,517	99.02	41,589	41,119	98.87	28,562	68.68	3599	8.65 (8.28–8.92)
2021	40,389	39,867	98.71	39,939	39,493	98.88	33,503	83.89	3152	7.89 (7.63–8.16)
2022	39,251	38,712	98.63	38,788	38,614	99.55	33,522	86.42	2953	7.61 (7.35–7.88)
Total	220,857	217,605	98.53	218,707	216,018	98.77	148,722	68.00	18,194	8.34 (8.22–8.45)
APC			0.13			0.30		18.88		-3.50
95%CI			−0.20, 0.46			0.13, 0.47		2.64, 37.68		−6.28, − 0.63
*P*			0.290			**0.012**		**0.033**		**0.031**

### Trends in HBV DNA testing and antiviral therapy among HBsAg positive pregnant women

As the provision of free routine DNA testing, antiviral treatment, and follow-up services for pregnant women with HBV infection in Baoan did not commence until November 2018, data for the year 2018 were not available. The coverage of HBV DNA testing among HBsAg positive pregnant women increased from 81.45% in 2019 to 90.62% in 2022, but this trend was not statistically significant (*P* = 0.095). As a result, an average of 20.97% of pregnant women were identified as high-risk. The coverage of high-risk women receiving antiviral therapy significantly increased from 63.59% in 2019 to 90.04% in 2022 (APC = 11.90, 95% CI 2.49 ~ 22.19, *P* = 0.031) (Table 2).

**Table 2:** Trends in coverage of HBV DNA testing and antiviral therapy in Baoan district

Year	Number of pregnant women with HBV infection (a)	Number of HBV DNA testing (b)	Coverage of HBV DNA testing (b/a, %)	^a^Number of high-risk women with HBV infection (c)	Proportion of high-risk women with HBV infection (c/b, %)	Number of ART among high-risk women (d)	Coverage of ART among high-risk women (d/c, %)
2018	4252	^b^NA	NA	NA	NA	NA	NA
2019	4238	3452	81.45	736	21.32	468	63.59
2020	3599	3171	88.11	552	17.41	426	77.17
2021	3152	2803	88.93	632	22.55	529	83.70
2022	2953	2676	90.62	552	20.63	497	90.04
Total	18,194	14,033	77.13	2943	20.97	2042	69.38
APC			3.35		1.61		11.90
95%CI			−1.42, 8.35		−21.49, 31.52		2.49, 22.19
*P*			0.095		0.815		**0.031**

^a^ High-risk women referred to those with HBV viral load higher than 2*10^6^ IU/ml during 2019–2020, and higher than 2*10^5^ IU/ml during 2021–2022

^b^*NA* Not Available

### Trends in immunoprophylaxis among HBV-exposed newborns

Between 2018 and 2022, a total of 18,396 live births in Baoan were exposed to HBV. The coverage of timely HBIG administration increased significantly from 97.74 to 99.30% (APC = 0.37, 95% CI 0.18 ~ 0.57, *P* = 0.009), the coverage of timely HepB-BD injection also increased from 98.44 to 99.43%, but the trend was not statistically significant (*P* = 0.074) (Table 3).

**Table 3:** Trends in coverage of HBIG administration and HepB-BD injection in Baoan district

Year	Number of HBV-exposed live births (a)	Total number of HBIG administration (b)	Overall coverage of HBIG administration (b/a, %)	Number of timely HBIG administration (c)	Coverage of timely HBIG Administration (c/a, %)	Total number of HepB-BD injection (d)	Overall coverage of HepB-BD injection (d/a, %)	Number of timely HepB-BD injection (e)	Coverage of timely HepB-BD injection (e/a, %)
2018	4285	4275	99.77	4188	97.74	4282	99.93	4218	98.44
2019	4286	4283	99.93	4223	98.53	4284	99.95	4249	99.14
2020	3633	3631	99.94	3587	98.73	3632	99.97	3608	99.31
2021	3197	3196	99.97	3168	99.09	3197	100.00	3189	99.75
2022	2995	2993	99.93	2974	99.30	2995	100.00	2978	99.43
Total	18,396	18,378	99.90	18,140	98.61	18,390	99.97	18,242	99.16
APC			0.04		0.37		0.02		0.26
95%CI			−0.03, 0.10		0.18, 0.57		0.01, 0.03		−0.05, 0.57
*P*			0.169		**0.009**		**0.005**		0.074

### Trends in coverage of PVST among HBV-exposed infants

HBV-exposed infants were followed up until 12 months of age, with data analyzed based on birth year. Only infants born from 2018 to 2021 were included, since not all 2022 births had concluded by the time of analysis.

Of the 15,401 HBV-exposed live births born between 2018 and 2021 (Supplementary Fig. S2), 23 died before 7 months of age, 13,190 (85.77%) were successfully followed up, showing a significant improvement in the follow-up rate from 80.43 to 92.33% (APC = 0.37, 95%CI 0.18 ~ 0.57, *P* = 0.009). HepB vaccination coverage remained consistently high (> 99.50%). However, only 7904 (59.96%) underwent PVST. Among the 2408 high risk infants for MTCT, only 1352 (56.15%) underwent PVST, with no significant trend difference over the years (*P* = 0.155) (Table 4).

**Table 4:** Trends in coverage of PVST and MTCT rate among HBV-exposed infants in Baoan district

Year	Number of infants that should be follow-up (a)	Number of followed-up (b)	Success rate of followed-up (b/a,%)	Number of three-dose HepB vaccine (c)	Coverage of three-dose HepB vaccine (c/b, %)	Total number of PVST (d)	Overall coverage of PVST (d/c, %)	Number of high risk infants for MTCT (e)	Number of PVST in high risk infants for MTCT (f)	Coverage of PVST in high risk infants for MTCT (f/e,%)	Number of HBsAg (+) (g)	MTCT rate of HBV (g/d,%)
2018	4278	3441	80.43	3441	100.00	2000	58.12	470	241	51.28	9	0.45
2019	4273	3645	85.30	3645	100.00	1836	50.37	742	349	47.04	3	0.16
2020	3632	3154	86.84	3149	99.84	2054	65.23	557	316	56.73	2	0.10
2021	3195	2950	92.33	2948	99.93	2014	68.32	639	446	69.80	0	0.00
Total	15,378	13,190	85.77	13,183	99.95	7904	59.96	2408	1352	56.15	14	0.18
APC			4.41		−0.04		7.72			11.77		−99.76
95% CI			1.76, 7.13		−0.18, 0.10		−14.15, 35.16			−9.78, 38.45	−100,1,183,000.74	
*P*			**0.019**		0.371		0.294			0.155		0.186

### Trends in MTCT rate of HBV and risk factors

Between 2018 and 2021, 14 cases tested positive for HBsAg, resulting in a MTCT rate of 0.18%. Although there was a decrease in the MTCT rate of HBV from 0.45 to 0%, the trend was not statistically significant (*P* = 0.186) (Table 4).

Multivariate logistic regression analysis revealed that HBV-exposed infants born to mothers with high school education or below faced a higher risk of HBV EMTCT than those born to mothers with college education or above (OR = 3.76; 95%CI 1.04 ~ 13.60, *P* = 0.04). Maternal HBeAg positivity was also identified as a risk factor for MTCT of HBV (OR = 18.89; 95%CI 1.98 ~ 18.50, *P* = 0.01) (Table 5).

**Table 5:** Univariate and multivariate analysis of factors associated with HBV infection among HBV-exposed infants in Baoan district

Factor	Total	HBsAg (+) N (%)	HBsAg (−) N (%)	*χ*^2^	*P*	adjusted OR (95%CI)
Maternal age (years)						
< 35	6706	14 (100.00)	6692 (84.82)	1.46	0.226	
≥ 35	1198	0 (0)	1198 (15.18)			
Maternal education level						
High school and below	3620	11 (78.57)	3609 (45.74)	6.07	**0.014**	**3.76 (1.04–13.60)**
College and above	4284	3 (21.43)	4281 (54.26)			1
Maternal residence						
Registered	2267	1 (7.14)	2266 (28.72)	2.21	0.137	
Non-registerd	5637	13 (92.86)	5624 (71.28)			
Maternal parity						
1	3489	6 (42.86)	3483 (44.14)	0.01	0.923	
≥ 2	4415	8 (57.14)	4407 (55.86)			
Mode of delivery						
Vaginal delivery	5175	10 (71.43)	5165 (65.46)	0.04	0.851	
Caesarean section	2729	4 (28.57)	2725 (34.54)			
Maternal HBeAg status						
Positive	2077	13 (92.86)	2064 (26.16)			**18.89 (1.98–180.52)**
Negative	5827	1 (7.14)	5826 (73.84)	28.74	**< 0.001**	1
Maternal HBV DNA viral load						
High	1356	10 (71.43)	1346 (17.06)	21.13	**< 0.001**	2.77 (0.52–14.73)
Low	4860	2 (14.29)	4858 (61.57)			1
Unknown	1688	2 (14.29)	1686 (21.37)			1.44 (0.19–10.65)
Maternal ART during pregnancy						
Yes	1498	2 (14.29)	1496 (18.96)	0.01	0.917	
No	6406	12 (85.71)	6394 (81.04)			
Infant gender						
Male	4340	8 (57.14)	4332 (54.90)	0.03	0.866	
Female	3564	6 (42.86)	3558 (45.10)			
Gestational age (weeks)						
< 37	490	1 (7.14)	489 (6.20)	/	^#^0.592	
37–43	7414	13 (92.86)	7401 (93.80)			
Child birth weight (g)						
< 2500	394	0 (0)	394 (4.99)	/	^#^1.00	
≥ 2500	7510	14 (100.00)	7946 (95.01)			

^#^ *Fisher’s* exact test

Furthermore, when comparing the low and high education groups among the 7904 HBV-exposed infants undergoing PVST, we observed significant differences in several indicators. The low education group had a higher proportion of HBeAg positive cases and high viral load, while they had a lower proportion of registered cases, systematic management, DNA testing, and antiviral treatment compared to the high education group (Table 6).

**Table 6:** Sub-group analysis of educational level among HBV-exposed infants undergoing PVST in Baoan district

Factor	Total	High school and below N (%)	College and above N (%)	*χ*^2^	*P*
Maternal residence					
Registered	2267	319 (8.81)	1948 (45.47)	1289.067	< 0.001
Non-registerd	5637	3301 (91.19)	2336 (54.53)		
^a^Maternal systematic management					
Yes	6408	2685 (74.17)	3723 (86.90)	207.322	< 0.001
No	1496	935 (25.83)	561 (13.10)		
Maternal HBeAg status					
Positive	2077	1028 (28.40)	1049 (24.49)	15.494	< 0.001
Negative	5827	2592 (71.60)	3235 (75.51)		
Maternal DNA testing					
Yes	6216	2686 (74.20)	3530 (82.40)	78.565	< 0.001
No	1688	934 (25.80)	754 (17.60)		
Maternal HBV DNA viral load					
High	1356	668 (18.45)	688 (16.06)	104.150	< 0.001
Low	4860	2018 (55.75)	2842 (66.34)		
Unknown	1688	934 (25.80)	754 (17.60)		
Maternal ART during this pregnancy					
Yes	1498	650 (17.96)	848 (19.79)	4.139	0.038
No	6406	2970 (82.04)	3436 (80.21)		

^a^Refers to meeting all the following requirements from pregnancy to 1 week after delivery: (1) had the first prenatal examination before 13 weeks of gestation, (2) had at least five prenatal examinations, (3) in-hospital delivery, and (4) had postnatal visit after delivery

## Discussion

This study highlights significant progress in the implementation of comprehensive interventions for HBV EMTCT in Baoan district. As of 2022, 86.42% of pregnant women received early HBV screening, and over 90% of high-risk infected pregnant women received ART. The coverage of timely administration of HBIG and HepB-BD exceeded 98%, and the rate of HBV MTCT reached a record low of 0% in 2021. All national targets for the validation of HBV EMTCT have been successfully achieved, except for the coverage of PVST among high risk infants for MTCT, which currently stands at 56.15%, falling below the the target of at least 90% (Supplementary Table S5).

Despite a decreasing trend in the prevalence of maternal HBV infection in Baoan district, the current prevalence rate of 7.65% in 2022 remained relatively high when compared to the national level of 5.44% in 2020 [6]. Our findings demonstrated that maternal HBV infection in Baoan fell within the higher intermediate endemic range, which could be attributed, in part, to the historically high prevalence of HBV infection in Guangdong province. National surveys conducted in 1979 [24] and 1992 [8] among the general population reported high rates of HBV infection during the time when the women in our study were born. Notably, however, our estimate is lower than the average prevalence rate of 9.84% in 2020 reported in Guangdong province [6], which had been a long-standing high endemic region for HBV infection. The high mobility of the population in Baoan, as observed in our study, where 42.62% of HBsAg positive pregnant women originated from other provinces, partly explained this finding. Our observation supported the notion of a mixed endemic mode observed at the provincial or county level [25].

In China, EMTCT has been integrated with ANC services for pregnant women, As part of this integration, free rapid HBV screening is provided at every maternal and child health institution. However, in Baoan district, there is a challenge as some pregnant women still opt out of the free HBV screening due to the merging of free and paid services that occurs after establishing their ANC records. To address this issue, Baoan introduced the policy of “screening first, ANC registration second” in May 2018, which aims to improve screening compliance among pregnant women by identifying infections early and provide timely intervention. As a result, our study found an annual increase of 18.88% in the coverage of HBV early screening from 2018 to 2022, which, in turn, led to an increase in coverage of ART among high-risk women.

High viral load HBV infection is often observed in HBeAg positive women and increases the risk of transmission, despite vaccine prophylaxis and HBIG [26]. Antiviral prophylaxis provision has been recommended as an additional protective measure for infants born to mothers with high HBV DNA levels [26]. In 2018, Chinese experts issued guidelines suggesting that pregnant women with a viral load ≥2*10^6^IU/ml be offered TDF from week 28 of pregnancy to further reduce the risk of vertical HBV transmission [22]. Later, the viral load threshold was lower to 2*10^5^IU/ml [27]. However, the HBV viral load testing rate among HBsAg positive pregnant women is very low, likely due to the cost of the test or the lack of clear testing requirements. According to the cooperative China-WHO pilot project conducted in eight counties in four provinces of China, the HBV DNA testing rate was < 1% among HBV infected mothers [28].

Baoan serves as a typical example where routine HBV DNA testing for pregnant women with HBV infection was not conducted prior to the initiation of the EMTCT pilot project. Furthermore, infected pregnant women had to be referred to the Hepatology department for treatment, leading to low treatment compliance and high loss to follow-up. To tackle these problems, Baoan established the E clinic within the obstetrics department of all midwifery hospitals in November 2018. This clinic operates as an integrated platform that combines the management of maternal infectious diseases with antenatal care, providing one-stop free-of-charge services for pregnant women infected with hepatitis B. The services offered include viral load testing, ART, guidance on delivery methods and breastfeeding, as well as regular antenatal check-ups and follow-up throughout the entire pregnancy. Notably, our study revealed a significant annual increase of 11.90% in ART coverage among high-risk women from 2019 to 2022, which can be attributed to the successful implementation of the E clinic service mode.

The E clinic model has been recognized as the best practice for EMTCT by the World Health Organization and China. It has also been included in the Training Hub of Maternal and Child Health (MCH) for Belt and Road Initiative (BRI) Countries, as well as the EMTCT Action Plan in Guangdong Province from 2023 to 2025. Since March 2023, this model has been promoted and implemented across the entire province, resluting in the esatblisment of the E clinic in 100 MCH institutions as of December 1, 2023.

The American Academy of Pediatrics recommends administering both HBIG and HepB-BD within 12 h of birth [29] to ensure timely HBV post-exposure prophylaxis. China updated its national guidelines in 2020 from within 24 h of birth to within 12 h [30]. In this aspect, Baoan has pioneered the “immediate immune next to bedside” strategy since January 2018. Delivery hospitals provide free HepB-BD and HBIG to newborns who were exposed to HBV immediately in the delivery room or operating room. This has resulted in 98.61% timely HBIG coverage and 99.16% timely HepB-BD coverage.

PVST is a valuable tool for determining the HBV infection status and confirming the efficacy of the hepatitis B vaccine in HBV-exposed children. It is noteworthy that both the WHO and the US Center for Disease Control and Prevention (CDC) presently recommend PVST for infants born to HBsAg positive mothers [31–33]. In China, PVST began in 2016 [34] and was subsequently integrated into the updated national iPMTCT guidelines in 2020 [23]. Baoan initiated free follow-up and PVST in May 2018. Unfortunately, the coverage of PVST for HBV-exposed infants in Baoan remained below 60%, which was lower than the reported 65.6% PVST coverage in four provinces of China in 2014 [35] and 67.08% in Zhejiang province between 2016 and 2020 [36].

It is noteworthy that, in contrast to PVST pilot provinces where refuse/failure of blood collection was reported as the primary cause of loss to follow-up (53.2%) [34], the lack of awareness of PVST among guardians (56.73%) was the main reason for non-compliance in Baoan district. Specifically, this refers to not knowing (44.71%), forgetting (10.68%) and not having enough time (1.34%) for the test. As PVST for HBV-exposed infants should be performed at no earlier than 6 months of age, the effectiveness of health education at the time of maternal discharge is greatly diminished due to the intervening period. This underscores the necessary for Baoan to focus on targeted publicity and education efforts aimed at raising awareness and ensuring compliance among guardians of HBV-exposed infants.

Our study findings support previous research by indicating that a lower maternal education level is a risk factor for MTCT of hepatitis B (OR = 3.76; 95%CI 1.04 ~ 13.60) [37]. This underscores the importance of targeted health education programs and interventions aimed at improving maternal knowledge and awareness of HBV transmission and prevention, particularly in vulnerable populations. However, it should be noted that low education level may not directly contribute to the risk but rather serve as an indicator for other underlying factors. These factors may include limited knowledge and awareness about MCH, restricted access to testing and treatment options, and socioeconomic pressures that impede optimal management of the infection. Therefore, caution must be exercised when interpreting our findings. Further research and comprehensive data collection are needed to gain a better understanding of the relationship between low education level and HBV MTCT.

Additionally, our study also indicated that infants born to HBeAg positive mothers are at greater risk of HBV MTCT (OR = 18.89; 95%CI 1.98 ~ 18.50). This discovery is consistent with previous studies that had established a correlation between maternal HBeAg positivity, higher levels of HBV replication, and intrauterine transmission of HBV [38]. The national guidelines for PMTCT of HBV (2020 edition) [23] recommend the use of HBeAg positivity as an alternative indicator for the administration of antivirals in HBsAg positive pregnant women. Therefore, HBeAg testing may be a convenient substitute for guiding ART in underdeveloped regions where routine HBV DNA testing may not be readily available.

### Limitation

Our study has several limitations. Firstly, some information such as the 2nd and 3rd doses of HepB vaccine and PVST was obtained through telephone follow-up, which may have resulted in information bias. Secondly, PVST outcomes were unavailable for roughly 40%, which may have led to underestimated EMTCT rate of HBV. Lastly, the implementation of the policy of free routine DNA testing and antiviral treatment in November 2018 resulted in a baseline difference for comparison. Consequently, the nine cases of MTCT identified in 2018 may have occurred in patients who did not receive antiviral treatment during pregnancy before the policy implementation.

## Conclusions

In summary, this study highlights the value and significant outcomes resulting from the implementation of a comprehensive prevention strategy targeting the reduction of Hepatitis B MTCT in Baoan district, China. The establishment of a regional, multi-institutional management mechanism and the introduction of the E clinic have provided valuable insights and practical evidence for other regions to learn from. However, challenges remain, especially regarding the suboptimal coverage of PVST for HBV-exposed infants. Further research is warranted to identify and implement targeted interventions and strategies for the ultimate goal of eliminating MTCT of Hepatitis B, not only in Baoan district, but also in other areas within China and globally.

### Supplementary Information

**Additional file 1.**

**Additional file 2.**

## Data Availability

Data is available upon reasonable request to the corresponding author.

## Abbreviations

anti-HBs: Antibody against HBsAg
anti-HBe: Antibody against HBeAg
anti-HBc: Antibody against hepatitis B core antigen
ANC: antenatal care
APC: Annual percentage change
ART: Antiviral therapy
BRI: Belt and Road Initiative
CDC: Center for Disease Control and Prevention
CI: Confidence interval
EPI: Expanded Programme on Immunization
ELISA: Enzyme-linked immunosorbent assay
EMTCT: Elimination of mother-to-child transmission
HBV: Hepatitis B virus
HBIG: Hepatitis B immunoglobulin
HepB-BD: Hepatitis B birth dose vaccine
HBsAg: Hepatitis B surface antigen
HBeAg: Hepatitis B e antigen
HIV: Human immunodeficiency virus
iPMTCT: Integrated prevention of mother-to-child transmission
IM: Immunological monitoring
LdT: Telbivudine
MCH: Maternal and Child Health
MTCT: Mother-to-child transmission
OR: Odd ratio
PMTCT: Prevention of mother-to-child transmission
PVST: Post-vaccination serological testing
TDF: Tenofovir Disoproxil Fumarate
WHO: World Health Organization
