# Supplementary material for: Eliminating mother-to-child transmission of hepatitis B virus: practice and progress in Baoan, a national pilot district of China
Source: BMC Public Health. 2024 Jan 2;24:58. doi: 10.1186/s12889-023-17500-y (PMC10763428; doi:10.1186/s12889-023-17500-y)
Supplement: Supplementary file 1 — Additional file 1. [file 12889_2023_17500_MOESM1_ESM.pdf]

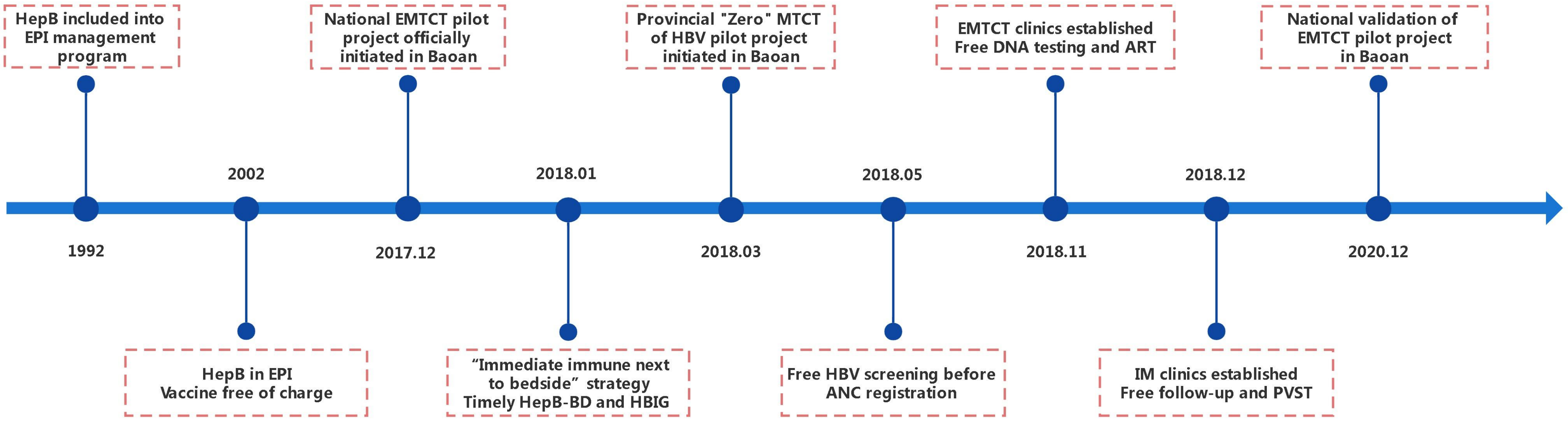

Figure S1 The progress of HBV EMTCT strategies in Baoan district

Note: *HepB* Hepatitis B, *EPI* Expanded Program on Immunization, *EMTCT* Elimination of Mother-To-Child Transmission, *HBIG* Hepatitis B Immunoglobulin, *HepB-BD* Hepatitis B Birth Dose vaccine, ANC Antenatal Care, IM Immunological Monitoring, ART Antiviral Therapy

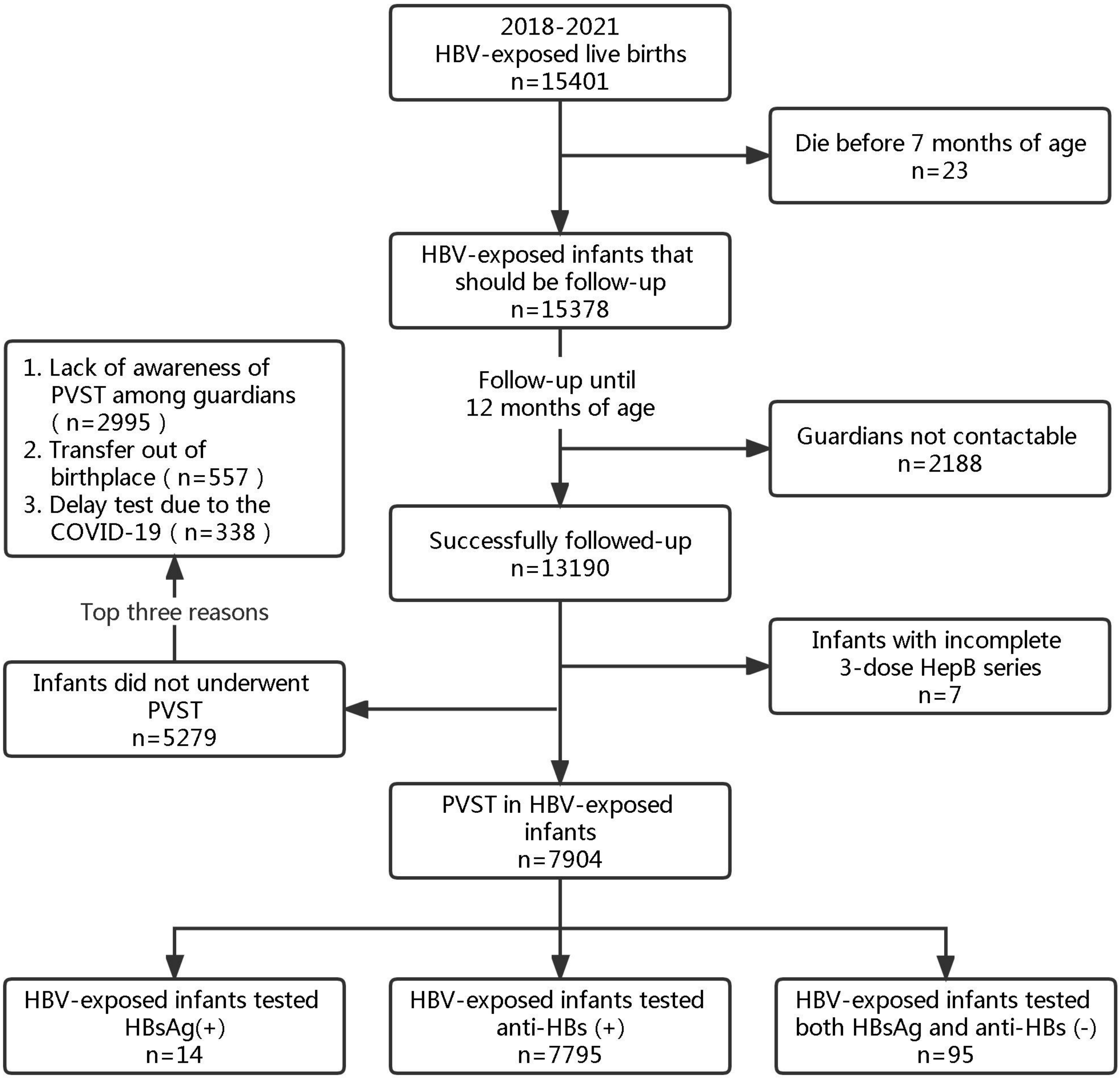

Figure S2 The included process of HBV-exposed infants who underwent PVST in Baoan district
