# Supplementary material for: Eliminating mother-to-child transmission of hepatitis B virus: practice and progress in Baoan, a national pilot district of China
Source: BMC Public Health. 2024 Jan 2;24:58. doi: 10.1186/s12889-023-17500-y (PMC10763428; doi:10.1186/s12889-023-17500-y)
Supplement: Supplementary file 2 — Additional file 2. [file 12889_2023_17500_MOESM2_ESM.pdf]

Supplementary Tables

Table S1 *Sheet 4-I Registration Form for pregnant women with HBV infection and their newborns*

______________Province (autonomous region, city)______________County (district)

1. Essential Information
Name:____ID number:_____________________________
Date of birth:_____year_____month_____day
Ethnic groups: ☐Han, ☐Zhuang, ☐Man, ☐Hui, ☐Miao, ☐Uygu, ☐Yi, ☐Tujia, ☐Mongolian, ☐Tibetan, ☐other:____________
Education level: ☐unknown, ☐illiterate/semi-illiterate, ☐primary school, ☐junior high school, ☐senior high school (including technical secondary school, vocational high school, technical school, etc.) ☐junior college or university, ☐master's degree or above
Occupation: ☐students (graduate student, university, middle school), ☐teacher, ☐nurse and nanny, ☐food and beverage industry, ☐business services, ☐medical staff, ☐workers, ☐farmers, ☐herdsmen, ☐fishing (boat) people, ☐cadres and staff, ☐retired personnel, ☐housework and unemployed, ☐others:____, ☐not quite clear
Marital status: ☐unmarried, ☐first marriage, ☐re-marriage, ☐cohabitation, ☐divorce, ☐widowed
Pregnancy and childbirth situation: Gravidity:______, Parity_____, Number of existing children:_____
Present address:_____economize_______market_____County (district)_______Township (town, street)______village_____(house number)
Registered permanent residence:___economize_____market___County (district)_____Township (town, street)____village____(house number)
The last menstrual time of this pregnancy: _____year_____month_____day
Contact number:_____________

2. Maternal hepatitis B virus-related testing situation (with + or-)

Test time and gestational age	Detection results					
	HBsAg	anti-HBs	HBeAg	anti-HBe	anti-HBc	HBV-DNA (IU/ml)
____weeks____day						
____weeks____day						
____weeks____day						

3. Maternal women receiving antiviral treatment (ART) during pregnancy:
☐ not receiving ART during pregnancy
☐ receiving ART during pregnancy, gestational age at the start of medication:____weeks____day
Medications: ☐tenofovir, ☐tibivudine, ☐lamivudine, ☐other:____________

4. Date of delivery: _____year_____month_____day, gestational age of delivery:____weeks____day

5. Mode of delivery: ☐natural delivery, ☐vaginal midwifery, ☐elective cesarean section, ☐emergency cesarean section, ☐unknown

6. Place of delivery: ☐municipal or above midwifery institutions, ☐county (district) level midwifery institutions, ☐township (sub-district) level midwifery institutions, ☐home, ☐other:_____________
Name of the delivery institution:____

7. Information of newborn infants (if there are multiple live births, please fill in the basic information and vaccination status of the infants in the attached sheet respectively)
Name:______________________Gender: ☐male, ☐female
Birth weight: ______gram, birth length:_____cm, time of birth: _____year_____month_____day____hour____minute
Survival: ☐survival, ☐death, and cause of death:____________________, time of death: _____year_____month_____day

8. The first dose of hepatitis B vaccine vaccination: ☐vaccination, ☐not vaccinated (after filling in the reason, jump to "9"), the reason:______
Time of vaccination: _____year_____month_____day____hour____minute, ☐unknown
Vaccine type: ☐recombinant yeast, ☐recombinant CHO, vaccination dose: ☐10g, ☐20g, ☐other:________________

9. Hepatitis B immunoglobulin injection: ☐injection within 12 hours, ☐>12 hours of injection, ☐without injection, the reason:____________
Time of injection:_____year_____month_____day____hour____minute, ☐unknown
Injection dose: ☐100 IU, ☐other:___________

Reporting institution (seal):___________________________________Reporter:___________________________________
Contact number:____Completion date: _____year_____month_____day

Table S2 *Sheet 4-II Follow-up Form for infants born to pregnant women with HBV infection*

_______________Province (autonomous region, city)_______________County (district)

1. The Child's name:_________, Gender: ☐male, ☐female, Date of birth: _____year_____month_____day, Contact number:_____________

Age:_____months, Date of Follow-up:_____year_____month_____day, Name of the follow-up staff:______________

2. Follow-up of children: (if there are multiple live births, please separate schedule)

(I) Name of the follow-up institution:____

(2) Follow-up: ☐not follow-up, ☐successful follow-up, ☐lost to follow-up, reason:_________

(Children not reported loss to follow-up until 15 months of age)

(3) Survival: ☐survival, ☐death, and cause of death:______________, time of death:_____year_____month_____day

(4) Growth and development: body weight: ☐unknown, or ☐______kilogram, Length: ☐unknown, or ☐______centimeter

(5) Hepatitis B vaccine vaccination status:

Agent times	Vaccination				
	whether or not vaccinate	Vaccination time	dosage of inoculation	Vaccine types	Vaccination institution
second dose	☐yes ☐no	☐___year___month___day ☐unknown	☐10g, ☐20g, or☐others:___	☐recombinant yeast ☐recombinant CHO	
third dose	☐yes ☐no	☐___year___month___day ☐unknown	☐10g, ☐20g, or☐others:___	☐recombinant yeast ☐recombinant CHO	
fourth dose	☐yes ☐no	☐___year___month___day ☐unknown	☐10g, ☐20g, or☐others:___	☐recombinant yeast ☐recombinant CHO	
Remarks	(Non-mandatory)____				

(6) Detection of serological markers of hepatitis B virus infection:

☐not tested, reason:______________________________, ☐testing, testing institution: __________________________

Testing time: _____year_____month_____day

Testing method: ☐enzyme-linked immunosorbent test, ☐chemiluminescent, ☐colloid gold, or ☐other:_________

Testing results: HBsAg: ☐positive, ☐negative, ☐unknown, ☐other:____________

Anti-HBs: ☐positive, __________mIU/ ml, ☐negative, ☐unknown, ☐other:__________

Reporting institution (seal):___________________________________Reporter:_________________________________

Contact number:____Completion date: _____year_____month_____day

Remarks: ____

Table S3 *The national validation indicators for the EMTCT of HBV (2022 edition)*

Indicator		Definition	Target
**Impact Indicator**			
1	MTCT rate of HBV	The proportion of infants born to HBsAg positive mothers who test positive for HBsAg within 12 months of age	≤1%
**Process Indicators**			
1	Coverage of ANC	The ratio of the number of women who received at least one ANC to the number of live births	≥95%
2	Coverage of HBV screening in pregnant women	The proportion of pregnant women who have undergone HBV screening	≥95%
3	Coverage of HBV early screening in pregnant women	The proportion of pregnant women who have undergone at least one HBV screening during early pregnancy (within 12^{+6} weeks of gestation)	≥70%
4	Coverage of timely birth dose HBIG	The proportion of infants born to HBsAg positive mothers who received timely (within 12h of birth) administration of HBIG	≥95%
5	Coverage of timely HepB-BD	The proportion of infants born to HBsAg positive mothers who received timely (within 12h of birth) injection of HepB-BD	≥95%
6	Proportion of three-dose HBV vaccine in HBV-exposed infants	The proportion of infants born to HBsAg positive mothers who completed the second and third dose HBV vaccine within 12 months of age	≥95%
7	Coverage of PVST in HBV-exposed infants at a high MTCT risk	The proportion of high-risk infants born to HBsAg positive mothers (maternal HBV DNA viral load $\geq 2*10^5$) who received PVST within 12 months of age after immunoprophylaxis	≥90%
8	ART rate for HBsAg positive pregnant women at a high MTCT risk	The proportion of HBsAg positive pregnant women at high risk of MTCT (maternal HBV DNA viral load $\geq 2*10^5$) who received ART	≥90%

HBV hepatitis B virus, *MTCT* mother-to-child transmission, *HBsAg* hepatitis B surface antigen, *ANC* antenatal care, *HBIG* hepatitis B immunoglobulin, *HepB-BD* hepatitis B birth dose vaccine , *PVST* post-vaccination serological testing, *HBeAg* hepatitis B e-antigen, *ART* antiviral therapy

Table S4 *Monthly Report on the Prevention of Mother-to-Child Transmission of HIV, Syphilis, and Hepatitis B*

( ) Year ( ) month

Province (autonomous region, municipality directly under the central government):________________________________

City (prefecture, state):______________________

County (district):___________________________

No.	Item			Numbers	
1	Pre-marital Testing	Number of individuals receiving pre-marital healthcare		Male:	Female:
2		Number of individuals receiving HIV testing		Male:	Female:
3		Number of individuals infected with HIV		Male:	Female:
4	Pregnancy Testing	Number of pregnant women receiving antenatal care for the first time			
5		Number of pregnant women receiving HIV testing			
6		Number of pregnant women infected with HIV/AIDS			
7		Number of pregnant women receiving syphilis testing			
8		Number of pregnant women infected with syphilis:			
9		Number of pregnant women receiving hepatitis B testing			
10		Number of pregnant women infected with hepatitis B			
11	Pregnatal testing and intervention	Number of women receiving inpatient delivery services			
12		HIV	Number of pregnant women receiving HIV testing during antenatal and postnatal period		
13			Number of pregnant women receiving HIV testing during early pregnancy		
14			Number of pregnant women with positive HIV test results during the early pregnancy		
15			Number of pregnant women receiving HIV testing only during childbirth		
16			Number of pregnant women with positive HIV test results only during childbirth		
17			Total number of pregnant women infected with HIV		
18			Number of live births by pregnant women infected with HIV		

19		syphilis	Number of pregnant women receiving syphilis testing during antenatal and postnatal period	
20			Number of pregnant women receiving syphilis testing during early pregnancy	
21			Number of pregnant women with positive syphilis test results during early pregnancy	
22			Number of pregnant women receiving syphilis testing only during childbirth	
23			Number of pregnant women with positive syphilis test results only during childbirth	
24			Total number of pregnant women infected with syphilis	
25			Number of live births by pregnant women infected with syphilis	
26		hepatitis B	Number of pregnant women receiving hepatitis B testing during antenatal and postnatal period	
27			Number of pregnant women receiving hepatitis B testing during early pregnancy	
28			Number of pregnant women with positive hepatitis B test results during early pregnancy	
29			Number of pregnant women receiving hepatitis B testing only during childbirth	
30			Number of pregnant women with positive hepatitis B test results only during childbirth	
31			Total number of pregnant women infected with hepatitis B	
32			Number of live births by pregnant women infected with hepatitis B	
33			Number of children receiving hepatitis B immunoglobulin injection	
34			Number of children completing the first dose of hepatitis B vaccine	
35	Number of non-hospitalized women giving birth			

Reporting Date: Responsible staff of reporting institution:

Reporter: Reporting institution (seal):

Note: The maternal and child health care institutions shall summarize the contents completed by the relevant institutions and fill in them.

Table S5 *Management of HBV ETCT in MCH Settings: Targets, Strategies, and Achievements*

Who	Where	What	Target	How（Strategy）
Pregnant women	18 Midwifery hospitals	HBV Screening	☑ Coverage of ANC ≥95%<br>☑ Coverage of HBV screening ≥95%<br>☑ Coverage of HBV early screening ≥70%	Free HBV screening before ANC registration
HBsAg(+) pregnant women	18 Midwifery hospitals	• HBV DNA testing<br>• ART for high-risk<br>• Follow-up	☑ ART rate among high MTCT risk ≥90%	E clinic<br>（One-Stop services）
Delivery → Sheet 4-I → Baoan Women’s and Children’s Hospital				
HBV-exposed newborns	18 Midwifery hospitals	• HepB-BD injection<br>• HBIG administration	☑ Coverage of timely birth dose HBIG ≥95%<br>☑ Coverage of timely HepB-BD ≥95%	Immediate immune next to bedside
HBV-exposed infants（within 6 months）	Over 140 Community health service centers	2nd and 3rd Vaccination	☑ Proportion of three-dose vaccination ≥95%	National Immunization Program
HBV-exposed infants（7-12 months）	18 Midwifery Hospitals	• PVST<br>• Follow-up	☒ Coverage of PVST among high MTCT risk ≥90%<br>☑ MTCT rate of HBV ≤1%	IM clinic
Closed → Sheet 4-II → Baoan Women’s and Children’s Hospital				
